# Real-time simultaneous monitoring of multiple analytes in bacterial cultures

**DOI:** 10.1128/aem.01810-25

**Published:** 2025-11-18

**Authors:** Maggie M. Fink, Zahra Aljuboori, Maggie K. Klaers, Morgan Underdue, Joseph Malkovsky, Shahir S. Rizk

**Affiliations:** 1 Department of Chemistry and Biochemistry, Indiana University South Bend 14686, South Bend, Indiana, USA; 2 Department of Biochemistry, Molecular Biology and Biophysics, University of Minnesota 200766 , Minneapolis, Minnesota, USA; 3 Department of Biochemistry and Molecular Biology, Indiana University School of Medicine 12250, Indianapolis, Indiana, USA; Kyoto University, Kyoto, Japan

**Keywords:** biosensor, multi-sensor array, bacterial periplasmic binding proteins, bacterial metabolism, fluorescence biosensor

## Abstract

Bacterial metabolites are essential for biological processes, influencing human health, ecosystems, and various industrial applications. Simultaneous real-time monitoring of these metabolites is critical in understanding microbial dynamics, particularly in bioreactors and food or drug manufacturing. Current approaches often rely on offline methods, which are labor-intensive and susceptible to contamination, or genetic engineering techniques limited to single-analyte monitoring. Here, we present a novel method utilizing engineered periplasmic binding proteins (PBPs) conjugated with fluorophores to track multiple metabolites simultaneously in bacterial cultures, including *Escherichia coli*, *Pseudomonas aeruginosa*, *Staphylococcus aureus*, *Enterococcus faecalis*, and *Clostridium striatum*. This system continuously monitors the levels of multiple analytes, such as glucose, arabinose, ribose, glutamate, and arginine, providing high temporal resolution while maintaining sensor stability over a period of 24 h. Our findings confirm hierarchical substrate utilization in *E. coli* and other bacterial species and demonstrate the versatility of PBP-based multi-sensor arrays. This approach offers a non-invasive, modular, and scalable tool for bacterial metabolite analysis, paving the way for advances in both fundamental discoveries and practical applications in microbiology.

**IMPORTANCE:**

Real-time monitoring of metabolites in bacterial cultures is crucial for advancing our understanding of microbial physiology, metabolic fluxes, and dynamic responses to environmental changes. This capability enables researchers to capture transient metabolic states that are often missed in endpoint measurements. The use of engineered periplasmic binding proteins as biosensors for this real-time metabolite monitoring represents a groundbreaking approach. By leveraging the natural specificity and high affinity of PBPs for small molecules, these biosensors can be engineered to detect a wide range of metabolites with exceptional sensitivity and temporal resolution. The integration of PBP-based biosensors into microbial research not only enhances our ability to study real-time metabolism but also provides a versatile tool for optimizing industrial bioprocesses and exploring bacterial infections and complex microbial ecosystems

## INTRODUCTION

Bacterial metabolites are crucial for various biological processes and significantly impact human health and the environment (1–5). These small molecules, produced or consumed during bacterial metabolism, can serve as essential nutrients, signaling molecules, or antimicrobial agents, influencing the microbial community and host physiology (6–14). In the human body, for example, gut bacteria produce metabolites that regulate immune function, maintain gut integrity, and influence brain health (4, 6, 7, 15, 16). Bacterial metabolites also play a vital role in ecosystems, contributing to nutrient cycling and supporting plant growth by promoting root development or protecting against pathogens (15, 17–20). Understanding and harnessing these metabolites can lead to advances in medicine, agriculture, and biotechnology (21).

The ability to identify and simultaneously track the levels of multiple metabolites in bacterial cultures is also important for bioreactors used in the food and drug industries (22). Sensors deployed in the complex environment of a growth medium must provide sufficient accuracy, specificity, and sensitivity. Additionally, the sensors must possess the stability to withstand the duration of the experiment or prep while having an appropriate response time. Currently, several types of spectroscopy, electrochemistry, and mass spectrometry are employed as reliable methods to identify a wide range of metabolites (23–30). But often, these methods require “off-line” monitoring, where samples are extracted from the culture at different time points for analysis. These “off-line” measurements can be time and labor-intensive and may result in introducing contamination to the cultures (22). While some real-time monitoring of metabolites in bacterial samples has been constructed, they rely on genetic engineering to introduce fluorescence reporters to bacterial strains which are time-intensive, not universally applicable, and are often limited to monitoring single metabolites or small molecules (31–35).

Here, we describe the use of engineered periplasmic binding proteins (PBPs) conjugated with various fluorophores to simultaneously detect multiple metabolites over time in *E. coli*, *P. aeruginosa*, *S. aureus*, *E. faecalis*, *and C. striatum* monocultures in various growth conditions. PBPs comprise a superfamily of receptor proteins that bind to and recognize a wide variety of metabolites ranging from sugars, amino acids, ions, metals, and even aromatic byproducts of lignin degradation (36, 37). Bacteria species use PBPs to scavenge molecules from the environment using a two-component system that regulates nutrient uptake, gene expression, and chemotaxis (38, 39). A characteristic feature of PBPs is that ligand binding is associated with a large conformational change through a hinge-bending motion, transitioning from an open, unbound to a closed, bound conformation (40, 41). Many PBPs have previously been engineered into biosensors by specific attachment of an environmentally sensitive fluorophore near the hinge region or the binding pocket (36, 42). As a result, the conformational change associated with binding triggers a change in fluorescence which can then be used to indicate changes in ligand concentration.

Our method utilizes PBPs to monitor several analytes in bacterial cultures continuously. We use PBP-fluorophore conjugates to detect single, double, or triple analytes while monitoring bacterial growth. Exogenous addition of the sensor proteins shows the ability to continuously monitor signals over 24 h in planktonic culture using a fluorescence plate reader, with changes in the signals corresponding to the depletion or production of the tested metabolites, glucose, arabinose, ribose, glutamate, arginine, and ornithine. We show that the engineered biosensors remain stable and sensitive over time, ensuring accurate monitoring of metabolites. Our findings provide a foundation for engineering protein-based, metabolite-specific multi-sensor arrays that can help track and quantify metabolites in complex mixtures without the need for sampling or strain-specific genetic approaches.

## RESULTS

### Characterization of PBP-based fluorescent biosensors

**Fig 1:**
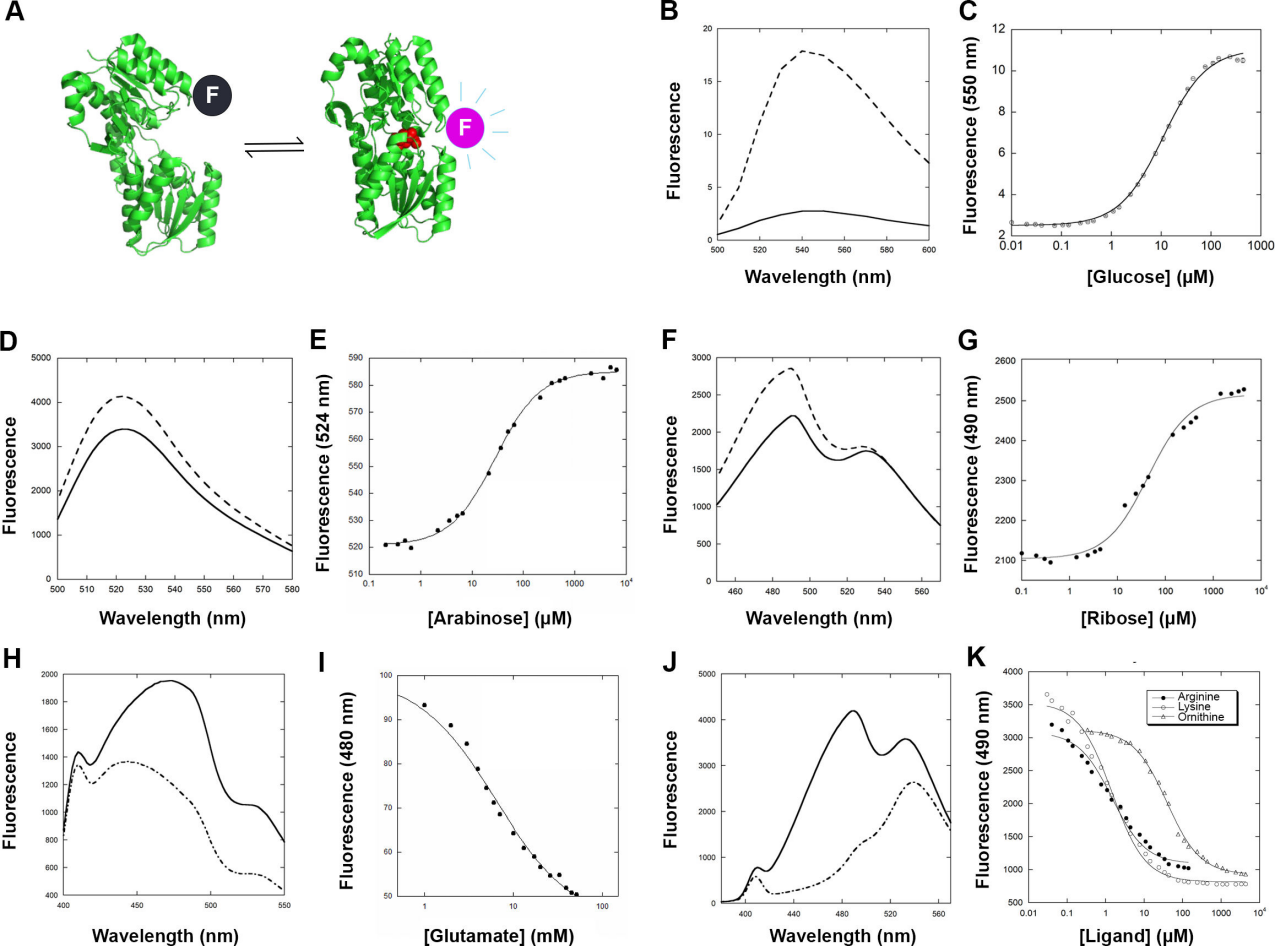
Characterization of PBP-fluorophore conjugates. (**A**) PBPs undergo a large hinge-bending conformational change upon binding their ligand (red). The change in fluorescence emission of a conjugated fluorophore “F” can be used as an indication of ligand binding. Shown here as an example is a thermophilic glucose binding protein (PDB codes: 3C6Q and 2H3H). (**B—K**) Fluorescence intensity of PBP-fluorophore conjugates in the absence (solid lines) and in the presence of ligand (dashed lines), followed by titration curves. TnGBP-IANDB (**B and C**), ABP-Alexa488 (**D and E**), TteRBP-Acrylodan (**F and G**), EBP-Coumarin (**H and I**), and LAOBP-Acrylodan (**J and K**).

To detect multiple analytes, we characterized the changes in fluorescence associated with ligand binding of several PBP-fluorophore conjugate combinations. We used glucose-binding protein (TnGBP) from *Thermotoga neapolitana* (43)*,* arabinose-binding protein (ABP) from *E. coli* (36)*,* ribose-binding protein (TteRBP) from *Thermoanaerobacter tengcongensis* (44), and aspartate/glutamate-binding protein (EBP) from *E. coli* (36) to detect changes in glucose, arabinose, ribose, and glutamate concentrations, respectively. All members of the PBP superfamily undergo a large hinge-bending conformational change upon ligand binding. Attaching a single fluorescent reporter near the hinge is an effective way to monitor binding by tracking changes in fluorescence (Fig. 1A). A single cysteine mutation in the protein is used to attach a thiol, reactive fluorescent reporter. A combination of previously published (36) and new cysteine mutations was introduced to the various PBPs for conjugation with fluorophores for the detection of changes in respective ligand binding.

Changes in fluorescence were determined by testing multiple fluorophores to determine the optimal signal range. For tnGBP, conjugation with IANBD (N,N'-dimethyl-N-(iodoacetyl)-N'-(7-nitrobenz-2-oxa-1,3-diazol-4-yl)ethylenediamine) resulted in a large increase in fluorescence emission upon glucose binding (Fig. 1B). Alexa488 and acrylodan gave an increase in fluorescence with ligand binding for ABP (Fig. 1D) and TteRBP (Fig. 1F), respectively. A decrease in fluorescence was observed for EBP conjugated with coumarin when bound to glutamate (Fig. 1H). The dissociation constant (*K*_d_) of each PBP for its ligand was determined by monitoring fluorescence change as a function of ligand concentration (Fig. 1). The *K*_d_ is defined as the midpoint of binding and reflects the affinity of the protein for its ligand. It also determines the linear range for detection, which is usually between 0.1× and 10× the *K*_d_. For example, tnGBP, which has a *K*_d_ of 11 µM,would reliably show changes in glucose concentrations between 1 and 110 µM (36). *K*_d_ values for the characterized PBPs described ranged between 11 µM and 6.4 mM (Table S1).

### Developing a fluorescent biosensor for arginine

Arginine and the structurally similar amino acid, L-ornithine, have recently been shown to be important metabolites for bacterial behavior, specifically in infection environments, serving as environmental signals (45–51). Because of this, we decided to design and develop a biosensor for arginine, using the lysine/arginine/ornithine binding protein from *Salmonella* (LAOBP) (52). We carried out cysteine scanning by introducing four individual cysteine mutations at positions near the hinge region. The selection of cysteine positions was guided by the crystal structures of the apo and bound forms of the protein (53) (Fig. S1). Each of the four mutants was conjugated to six different fluorophores, and the fluorescence change upon the addition of ligand was characterized for each of the 24 protein-ligand conjugates. Of the four mutants, only the LAOBP-22C mutant showed changes in fluorescence >10% with four of the six fluorophores tested (Table 1). We determined the dissociation constants for all three ligands (arginine, lysine, and ornithine) using the LAOBP-22C acrylodan conjugate (Fig. 1F) and the coumarin conjugate (Fig. S2). With a large change in fluorescence and a dissociation constant of 1.5 µM for both arginine and lysine, we chose to use the LAOBP-22C acrylodan conjugate for subsequent experiments.

**TABLE 1:** Characterization of the LAOBP-22C mutant conjugated with different fluorophores^*a*^

Fluorophore	% Change	*Kd* (µM)		
		Arginine	Lysine	Ornithine
Acrylodan	−50	1.5 ± 0.1	1.5 ± 0.1	38.9 ± 1.8
Coumarin	−53	2.2 ± 0.2	2.2 ± 0.2	51.4 ± 4.1
Alexa488	+8	ND	ND	ND
IANBD	-8	ND	ND	ND
Texas Red	−22	ND	ND	ND
TMR	−10	ND	ND	ND

^*a*^ ND, not determined.

### Temporal monitoring of single analytes in bacterial cultures

**Fig 2:**
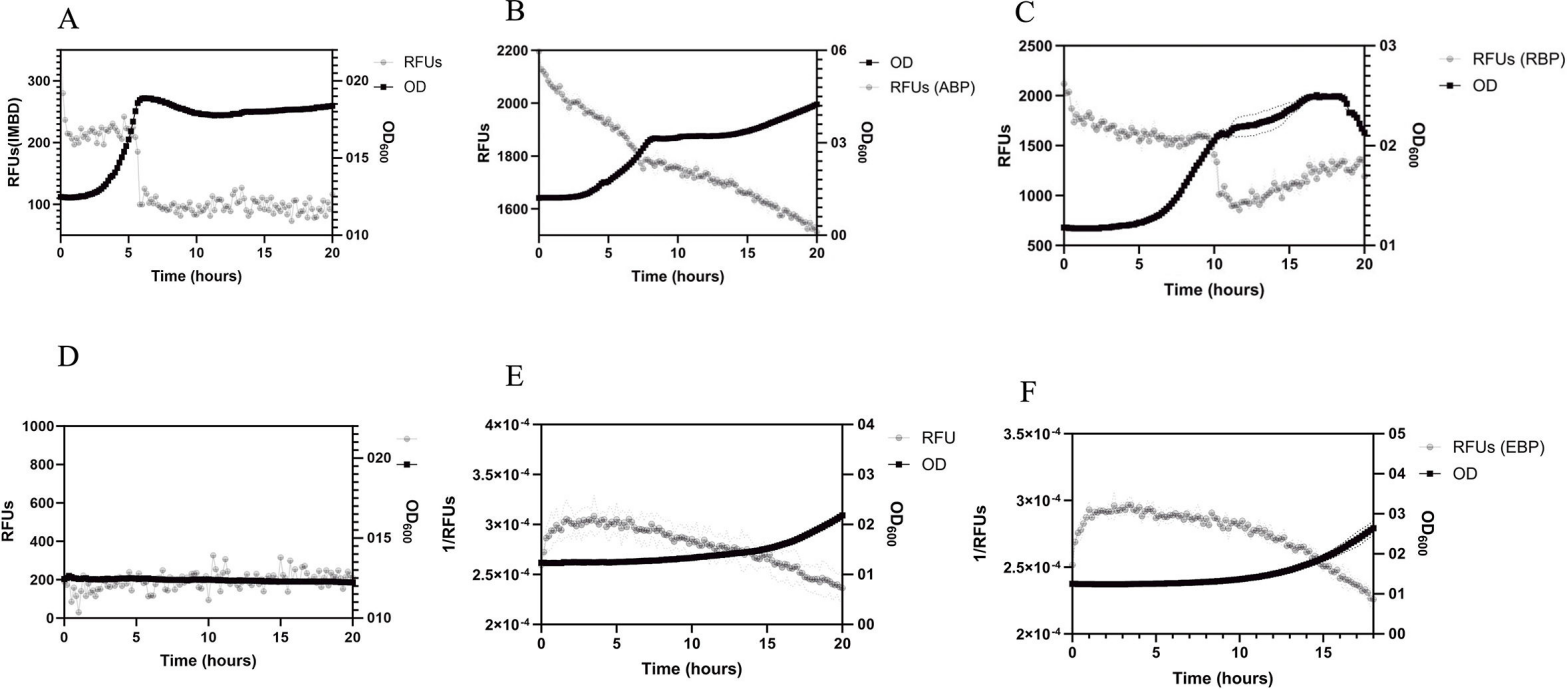
Engineered PBP-based biosensors can monitor metabolites in *E. coli* planktonic growth. *E. coli* was grown in 96-well plates in minimal media containing 1 mM of either glucose (**A**), arabinose (**B**), ribose (**C**), arginine (**D**), ornithine (**E**), or glutamate (**F**). 100 nM of the corresponding labeled PBPs—tnGBP-IANBD, ABP-Alexa488, TteRBP-acrylodan, LAOBP-acrylodan, and EBP-Coumarin—was added to each culture. OD600 and fluorescence were measured every 10 min. The change in fluorescence (RFUs; left y-axis) corresponds with *E. coli* growth (OD600; right y-axis) and depletion of each metabolite, except for arginine, which is unable to be utilized as a carbon source.

To determine if the PBPs could be used to continuously monitor analytes in complex mixtures over time, 100 nM of fluorescently labeled PBPs were added to *E. coli* K12 cultures grown in minimal media containing defined carbon sources. Two hundred microliters of this mixture was added to each well in a 96-well plate. Each condition was done in triplicates. Additionally, media containing *E. coli* only and PBP only were also included in triplicate on the plate as controls. Fluorescence from wells containing *E. coli* only was used to subtract any background fluorescence. In media with glucose (1 mM) and IANBD-labeled TnGBP, fluorescence sharply decreased over a period of 20 min corresponding to the mid-log phase of *E. coli* growth when glucose uptake and utilization occur rapidly (2, 54, 55) (Fig. 2A). During growth on arabinose (1 mM), the change in ABP-Alexa488 fluorescence was more gradual, which was consistent with the observed corresponding delay in log phase (Fig. 2B). Cultures grown on ribose (1 mM) in the presence of Acrylodan-labeled TteRBP showed a rapid decrease in fluorescence during log-phase growth, similar to glucose condition (Fig. 2C). Acrylodan-labeled LAOBP added to media containing only arginine (1 mM), which is not a sufficient carbon source for *E. coli* (56), did not exhibit any change in fluorescence or OD (Fig. 2D). However, the use of ornithine (1 mM), which can be utilized to some extent by *E. coli* (57)*,* resulted in a fluorescence change as OD increased at around 18 h (Fig. 2E). Similarly, in media containing only glutamate (1 mM), which is a poor carbon source (57), and EBP-Coumarin, *E. coli* growth was delayed, but a change in fluorescence was observed corresponding to an increase in OD (Fig. 2F). These results indicate that PBPs can detect the changes in corresponding individual analytes in real time during *E. coli* growth and reflect current understanding of metabolite uptake and utilization.

### Simultaneous monitoring of multiple analytes in bacterial cultures

**Fig 3:**
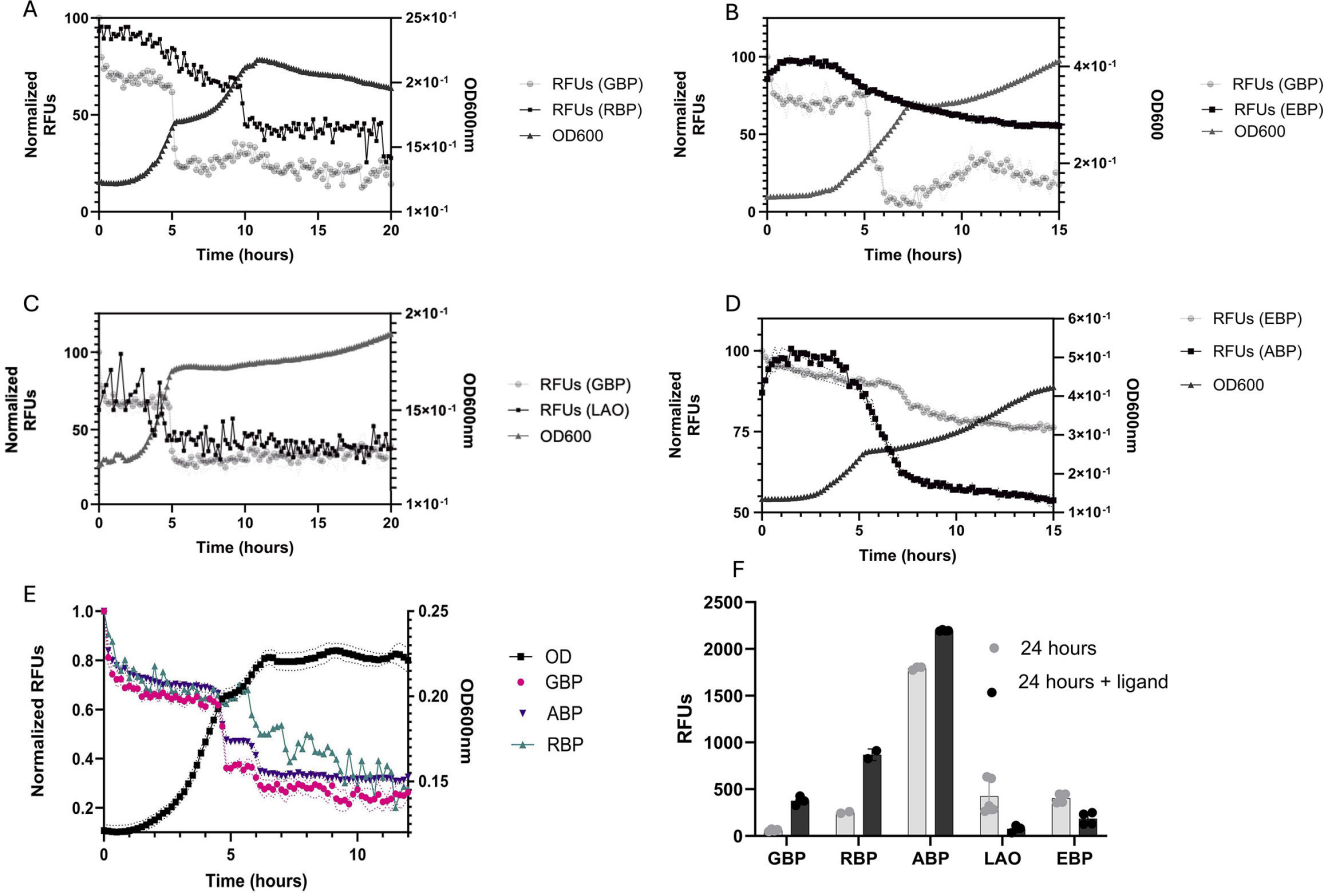
Multiple metabolites can be monitored simultaneously with PBPs during *E. coli* growth. *E. coli* was grown in minimal media containing 1 mM of glucose and glutamate (**A**), arabinose and glutamate (**B**), glucose and ribose (**C**), glucose and arginine (**D**), and glucose, arabinose, and ribose (**E**), with 100 nM of each corresponding labeled PBPs, tnGBP-IANBD, ABP-Alexa488, TteRBP-acrylodan, LAOBP-acrylodan, and EBP-Coumarin. Fluorescence for each biosensor was measured every 10 min as well as OD600. Changes in fluorescence indicating metabolite consumption are consistent with *E. coli’s* known hierarchical carbon utilization, indicating the metabolite-specific activity of each biosensor. (**F**) Ligands corresponding to each PBP, glucose, ribose, arabinose, arginine, and glutamate were added directly to cultures, and fluorescence was measured again. Change in fluorescence (increase or decrease) is consistent with Fig. 1, indicating that PBPs are still active after 24 h in *E. coli* culture and that changes in fluorescence are a result of analyte concentration in media.

*E. coli* was also grown in minimal media with different combinations of carbon sources and corresponding PBPs to monitor multiple analytes simultaneously. We grew *E. coli* on glucose and arginine, glucose and ribose, glucose and glutamate, glutamate and arabinose, or glucose, arabinose, and ribose (Fig. 3A through D). For each of the conditions, PBPs were able to detect changes in their corresponding analytes as they were depleted from the media. Our results are consistent with known hierarchical carbon utilization in *E. coli* (58–63). Specifically, in media with glucose and ribose, diauxic growth of *E. coli* (64) was observed with changes in fluorescence signals from tnGBP and TteRBP corresponding to two different exponential growth phases (Fig. 3A). In media containing glucose and glutamate, *E. coli* growth was slightly delayed; however, the decrease in tnGBP fluorescence corresponded with log phase, while EBP signal showed a gradual decrease as glutamate was utilized by *E. coli* (65, 66) (Fig. 3B). While LAOBP did not show any change in fluorescence when arginine was the sole carbon source (Fig. 2D), when glucose was added, LAOBP fluorescence change correlated with an increase in *E. coli* growth as glucose was depleted from the media (56) (Fig. 3C). Cultures growing on arabinose and glutamate (Fig. 3D) showed a similar trend to what was observed in media with glucose and glutamate. Both conditions showed a delayed log phase and changes in fluorescence corresponding to *E. coli* growth.

Finally, *E. coli* was grown in media with three different sugars: glucose, arabinose, and ribose along with their corresponding PBPs (Fig. 3E). Decreases in fluorescence were observed for each of the PBPs, again corresponding to *E. coli* diauxic growth phases known to occur on mixed carbon substrates (5, 58–61). The change in fluorescence was also sequential with tnGBP decreasing first, followed by ABP and finally TteRBP, which is consistent with previous studies characterizing hierarchical carbon utilization in *E. coli* (58, 60, 61).

### Monitoring nutrient consumption in complex media with different species

**Fig 4:**
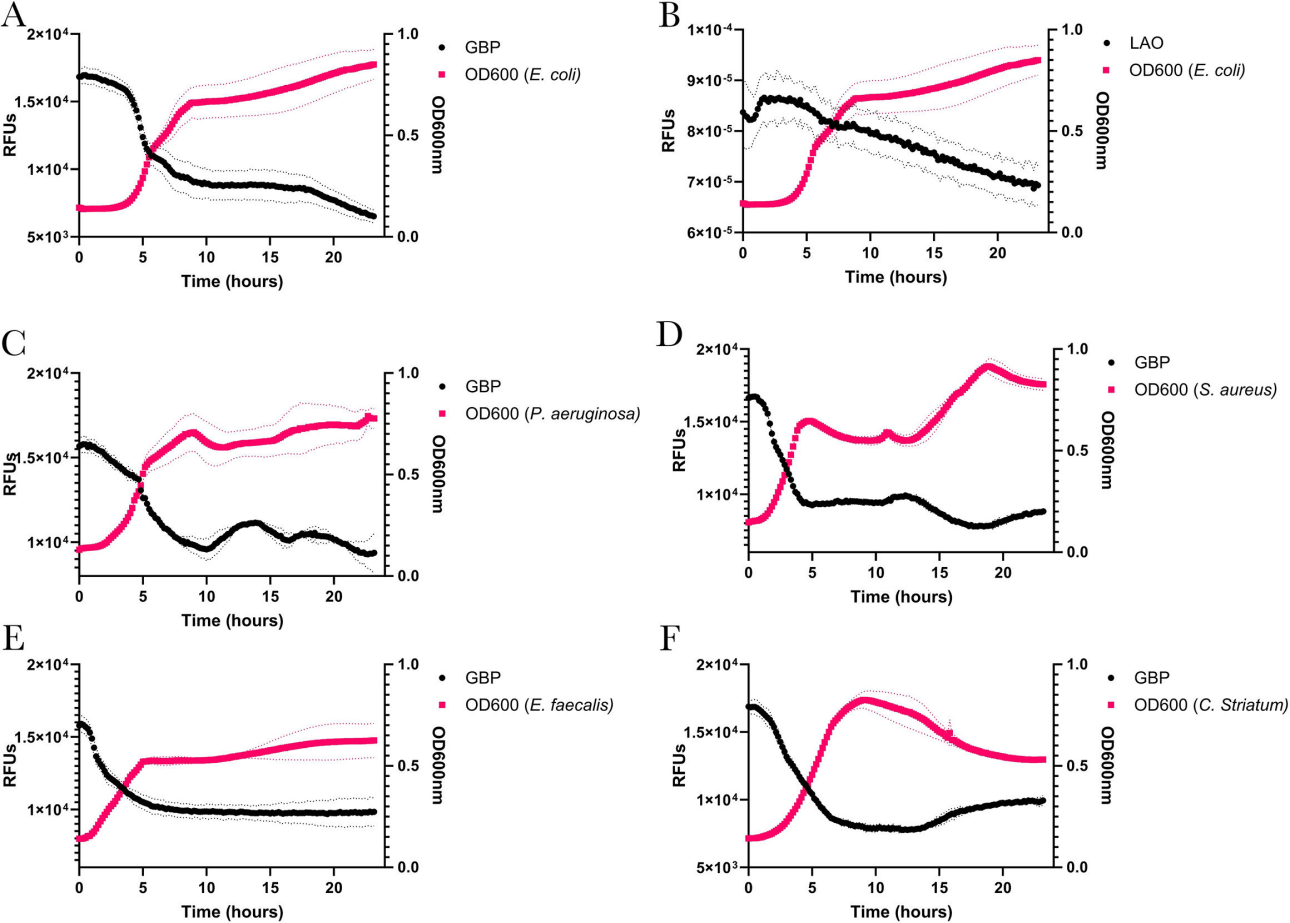
Engineered PBP biosensors can monitor metabolites of multiple bacterial species grown in rich media. *E. coli* (**A and B**), *P. aeruginosa* (**C**), *S. aureus* (**D**), *E. faecalis* (**E**)*,* and *C. striatum* (**F**) were grown in 200 µL of LB broth in 96-well plates. 100 nM of tnGBP-IANBD was added to each culture, and OD_600_ and fluorescence were measured every 10 min. OD_600_ corresponds with growth (right y-axis) of each species, and change in fluorescence (RFUs; left y-axis) indicates depletion of glucose during bacterial growth. The differences in growth and glucose consumption vary between each species; however, tnGBP-IANBD is able to detect changes in glucose concentrations even in complex media with specificity comparable to minimal media conditions. LAOBP-acrylodan was also tested in LB broth to monitor changes in arginine/ornithine during *E. coli* growth (**B**).

*E. coli* K12 (67) was grown in Luria-Bertani (LB) media with 1 mM of glucose added to determine if PBPs could monitor analytes in a complex environment. Cultures were again grown in 96-well plates with the previously described controls to account for background fluorescence. Like our results in minimal media, changes in normalized fluorescence for tnGBP decreased during *E. coli* growth (Fig. 4A), indicating that the engineered PBP is sensitive enough to detect changes in extracellular glucose in complex growth conditions. Similarly, the changes in normalized fluorescence for LAO were consistent with what is understood specifically about the metabolism of arginine. Early in the growth phase, arginine is produced to accommodate protein synthesis during rapid growth. As growth progresses toward the stationary phase, the expression of arginine biosynthetic genes typically decreases, coinciding with the accumulation of arginine both inside and outside the cell (56, 68, 69), which corresponds to the decrease in normalized fluorescence signal throughout the rest of *E. coli* growth (Fig. 4B).

In addition to *E. coli* K12, we selected multiple other bacterial species to test the performance of the biosensors. We tested the biosensors’ ability to monitors nutrients in cultures containing *Pseudomonas aeruginosa* PAO1C (70), *Staphylococcus aureus* USA300 (71), and clinical isolates *Enterococcus faecalis* EfPJI-A5 (72) and *Clostridium striatum* CsPJI-A24. Each species was grown in LB spiked with 1 mM of glucose. For tnGBP, the change in normalized fluorescence was similar to that measured in *E. coli*, with the largest changes coinciding with log-phase growth for each species; however, differences in the temporal changes between species also indicate that, in rich media, PBPs can detect changes in extracellular analytes that differ between the tested bacteria species (Fig. 4C through F). For example, *P. aeruginosa* and *S. aureus* both exhibited diauxic growth in LB, which corresponded to two separate changes in tnGBP fluorescence, while *E. faecalis* and *C. striatum* had a single decrease in fluorescence corresponding to the log phase, followed by a steady fluorescence signal during stationary phase.

### Stability of the biosensors during bacterial growth

To determine if the observed changes in fluorescence were not simply the result of degradation of the protein sensors over the course of *E. coli* growth, glucose, individual ligands (arabinose, ribose, glutamate, arginine, or ornithine) were added to 24-hour-old cultures containing each corresponding PBP. Immediately after the addition of the respective analyte, fluorescence was measured again (Fig. 3F). Each of the PBPs showed a change in fluorescence, indicating that the sensors are still active, and the changes observed over time in bacterial culture are a result of changes in analytes present in the media. In addition, gel electrophoresis of the media containing the biosensors showed that the proteins remained intact with no significant degradation throughout the 24-hour duration of the experiments regardless of the bacterial species used (Fig. S3). Our results show that the biosensors described here are robust, resistant to degradation by multiple species of bacteria, and remain active throughout the duration of the experiment.

## DISCUSSION

The method described here represents a non-invasive way to continuously monitor multiple nutrients simultaneously in bacterial cultures. By employing the specificity of PBPs and their characteristic conformation change upon ligand binding, each PBP acts as an independent sensor in a multisensor array, providing real-time information on how each nutrient is consumed. Other methods, such as mass spectrometry, require the collection of time points, which can be labor-intensive and can potentially introduce contamination. Our method bypasses the need for collecting time points, allowing high temporal resolution within minutes over hours or days.

The use of PBPs as the biosensor elements in the multisensor array described here offers several advantages. First, the superfamily of PBPs offers a wide repertoire of ligand specificity, making this method modular and expandable to include a wide variety of molecules—not only those consumed by bacteria, but also others that are produced and serve as signaling molecules. Furthermore, PBPs offer exquisite specificity for their cognate ligands. The vast majority of PBPs bind to either one molecule or a small set of related molecules. For example, glucose-binding protein is extremely specific for glucose, even replacing an -OH group at the 3′ position with a hydrogen results in a 100-fold decrease in affinity (43).

With binding kinetic constants in the sub-millisecond timescale (73), PBPs can quickly respond to a change in ligand concentration, providing high temporal resolution. Second, careful cysteine scanning provides multiple potential positions within each protein where one of several fluorophores can be attached, each showing a significant change in fluorescence upon binding to the ligand. This allows a mix-and-match approach, where different PBPs can be combined in several ways to detect different combinations of ligands with each PBP acting as a unique channel. Third, we show here that the PBP-fluorescent conjugates are stable and retain their ligand-binding capabilities for the entire duration of bacterial growth. Utilizing thermophilic PBPs offers an additional advantage for use under harsh conditions where other protein-based biosensors may become denatured. Finally, PBPs are highly engineerable. Mutations near and within the binding pocket can be introduced to fine-tune affinity of the protein for its ligand, allowing a tunable range for ligand detection (74). Furthermore, adding conformation-specific antibodies can also modulate the affinity of PBPs by controlling the equilibrium of the conformational change (75, 76). In some cases, PBPs have been modified to bind to different ligands (77) or entirely re-engineered to bind synthetic molecules (78), further expanding their application in real-time sensing.

For each of the engineered PBPs, we demonstrated their ability to be used as real-time monitors of metabolites over 24 h of *E. coli* growth. Using both single-carbon growth conditions and more complex media, such as LB, changes in fluorescence over time were monitored that coincided with changes in bacterial growth as each metabolite was being consumed or produced. In addition to monitoring metabolites in *E. coli* cultures, four other bacterial species were used: *P. aeruginosa*, *S. aureus*, *E. faecalis*, and *C. striatum*. Each of these showed similar changes in fluorescence during growth as was observed in *E. coli*, with minor differences corresponding to each species-specific carbon utilization strategies.

Here, we have also shown that these PBPs can be used in combination to simultaneously track changes in more than one analyte. Using combinations of sugars and amino acids, we were able to measure fluorescence of two PBPs at a time, with changes in fluorescence being observed during specific stages of *E. coli* growth. We also demonstrated the ability to detect the sequential consumption of three different sugars while monitoring the growth of *E. coli* using a fluorescent plate reader to measure fluorescence and OD_600_. Our data show, with high temporal resolution, that when presented with three different sugars, glucose is consumed first, followed by arabinose and then ribose. Without a parallel quantification method, our findings are consistent with known regulation mechanisms of *E. coli’s* sequential utilization of carbon sources (58, 60, 61). Additionally, these engineered PBPs maintain their specificity and resolution in more complex media, such as LB, which could allow for the detection of metabolites in a variety of environmental conditions. Using the method described here can contribute significantly to understanding bacterial metabolism and can help decipher the role of different metabolites in bacterial growth, communication, and pathogenesis (19, 79, 80).

Traditional methods for measuring metabolites in bacterial cultures, such as LC-MS and GC-MS, provide high analytical sensitivity and broad metabolite coverage (81, 82). These targeted LC–MS/MS workflows are highly sensitive: modern, well-validated targeted LC–MS assays routinely reach low-nanomolar limits of detection and quantification for many small metabolites, with reported LODs as low as ~1.4 nM for panels of serum/plasma metabolites (83). Many targeted amino acid assays report limits of quantitation around 0.1–1 µM (84, 85), and sugar assays often reach detection limits between 0.02 and 0.4 µM (86). While these techniques provide broad chemical coverage and excellent sensitivity, enabling the detection of trace metabolites and discovery work, they require quenching, extraction, and instrument run time for each discrete time point (87–89). This makes continuous, temporal resolution impractical and introduces sampling perturbation. They provide detailed but static “snapshots” of metabolite pools rather than continuous monitoring of dynamic changes occurring during bacterial growth or interspecies interactions.

While the method described here offers several advantages, there are still some limitations. For example, while ideal for on- and off-line temporal monitoring of analytes in culture, the method is semi-quantitative and limited to the linear range of detection based on the *K*_d_ values of each biosensor within two orders of magnitude. However, PBPs can be modified to change their range of detection, and in some cases, multiple mutants of the same PBP can be combined to expand the dynamic range of detection for a single analyte up to five orders of magnitude (90). Therefore, our results provide the first step toward a reliable method for simultaneously monitoring the changes in concentrations of analytes and nutrients in monocultures. Our results also provide the potential for studying bacterial metabolism beyond monocultures. By adapting this approach, it can be applied to multispecies cultures with the goal of understanding the temporal dynamics of interspecies bacterial communication mediated through chemical signals (91, 92). This has far-reaching implications in understanding infections, biofilm formation, the mechanisms of antibiotic resistance, and how commensal species compete or cooperate within host environments (93–96). Furthermore, with some modifications, this method could be extended to study nutrient uptake and metabolite release in a wide range of infection models, where altered metabolic pathways create unique nutrient environments that impact infection progression and severity.

## MATERIALS AND METHODS

All reagents, buffers, and salts were purchased from Thermo-Fisher unless otherwise indicated.

### Cloning and site-directed mutagenesis

The gene coding for TnGBP was cloned from *Thermotoga neapolitana* genomic DNA (ATCC) by PCR using primers that introduce an N-terminal NdeI site and a C-terminal NheI site. Genes for LAOBP, EBP, TtRBP, and ABP were ordered from IDT. PCR was used to amplify the genes using universal primers that introduce an N-terminal NdeI site and C-terminal NheI site. All genes were cloned into pET25b + cut with NdeI and NheI-HF in-frame with the plasmid sequence, which introduces a C-terminal HSV tag followed by a 6×His tag. Cloning was confirmed by DNA sequencing.

### Protein expression and purification

Plasmids coding for each of the proteins were used to transform BL21-DE3 *E. coli* cells by heat shock (45 sec, at 42°C) and plated on ampicillin LB agar plates. Single colonies were used to inoculate overnight cultures of 2XYT-amp, which were used to inoculate 250 mL 2XYT-amp in 1 L baffled flasks. After reaching OD600 of ~0.6–0.8, protein expression was induced by IPTG (1 mM final concentrations), and cultures were allowed to grow for an additional 3 to 4 h at 37°C or overnight at 16°C. Cells were isolated by centrifugation, resuspended in 20 mM TRIS-HCl, 500 mM NaCl, 10 mM imidazole, pH 8.6 (buffer A), lysed by addition of lysozyme and sonication in the presence of DNAse, and then centrifuged at 8,000 rpm for 30 min. The cleared lysate was loaded on a 1 or 5 mL His-Trap nickel-charged column (Cytiva) equilibrated with buffer A on an ÄKTA start. Proteins were isolated using a 25 mL gradient between buffer A and buffer B (20 mM TRIS-HCl, 500 mM NaCl, 10 mM imidazole, pH 8.6). SDS-PAGE analysis was used to identify fractions containing pure protein. Econo-Pac 10DG columns (Bio-Rad) were used to exchange the protein into working buffer (50 mM MOPS, 150 mM NaCl, pH 6.9).

### Fluorophore conjugation

A 5- to 10-fold excess of fluorophore to protein ratio was used to attach thiol-reactive fluorophores (dissolved in DMSO) to the cysteine residue of each of the PBPs. Reaction was carried out at room temperature overnight in the presence of TCEP (1 mM final concentration) tumbling end to end. Excess fluorophore was removed using Econo-Pac 10DG columns equilibrated with working buffer.

### Fluorescent screening and data analysis

Fluorescence analysis of protein-fluorophore conjugates was carried out on a Jasco FP-8500 fluorometer. UV-clear, four-sided cuvettes containing 200–300 nM labeled protein in working buffer were used. The following parameters were used for each of the fluorophores: Acrylodan (excitation: 359 nm, emission: 440–550 nm), Coumarin (excitation: 390 nm, emission: 430–520 nm), IANBD (excitation: 460 nm, emission: 500–600 nm), Alexa488 (excitation: 490 nm, emission: 500–560 nm), TMR (excitation: 555 nm, emission: 570–620 nm), and Texas Red (excitation: 595 nm, emission: 600–660 nm). Titrations were carried out by adding increasing amounts of ligand while monitoring changes in emission at the maximum wavelength. Fluorescence values were volume adjusted, and *K*_d_ values were determined by fitting the titration data using Kaleidagraph according to the following equation:


$$F=F_{o}+\frac{F_{max}-F_{o}}{1+\frac{K_{d}}{\left[L\right]}}$$

where *F* is the observed fluorescence at a given ligand concentration [*L*], *F*_o_ is the fluorescence in the absence of ligand, and *F*_max_ is the fluorescence at saturation.

### Bacterial strains and culture conditions

All bacteria strains used in this study are listed in Table S1. Cultures were streaked from frozen (−80°C) stocks onto Luria-Bertani broth (Sigma-Aldrich) agar plates (1.0% [wt/vol]) and incubated at 37°C overnight. Isolated colonies were selected and inoculated in a 6 mL LB. Tubes were incubated overnight at 37°C with shaking at 240 rpm. Cells were centrifuged at 10,000 rpm for 20 min, washed with 6 mL PBS, washed a second time, and then resuspended in 6 mL FAB medium with 1 mM carbon source. LB broth or FAB was used unless otherwise described.

### Growth curves

Growth curves were generated in clear 96-well plates (Corning Incorporated) and incubated at 37°C in a PowerWaveX 340 (BioTek Instruments, inc). Cultures were inoculated into 1 mL of LB broth (Sigma-Aldrich) or FAB to an OD_600_ of 0.01. 200 µL/well was added to 96-well black clear bottom plates (Corning Incorporated) for each culture condition, and absorbance at 600 nm was measured every 10 min for 24 h at 37°C. The plate was shaken for 10 s before each read. 100 nM of biosensor was added to each culture condition. Relative fluorescent intensity was captured every 10 min for 24 h at 37°C. The plate was shaken for 10 s before each read (all the excitation and emission values are listed in the “Fluorescence Analysis” subsection above).

Raw data from the plate reader was normalized by subtracting background fluorescence measured in cultures containing no PBP. For cultures containing more than one PBP to monitor multiple analytes, raw fluorescence data were normalized as previously described in addition to being represented by fold change.

## SUPPLEMENTAL MATERIAL

Supplemental tables and figures aem.01810-25-s0001.docx Tables S1 to S3, and Figures S1 and S2.

ASM does not own the copyrights to Supplemental Material that may be linked to, or accessed through, an article. The authors have granted ASM a non-exclusive, world-wide license to publish the Supplemental Material files. Please contact the corresponding author directly for reuse.
