# Supplementary material for: Real-time simultaneous monitoring of multiple analytes in bacterial cultures
Source: Appl Environ Microbiol. 2025 Nov 18;91(12):e01810-25. doi: 10.1128/aem.01810-25 (PMC12724168; doi:10.1128/aem.01810-25)
Supplement: Supplemental tables and figures — Tables S1 to S3, and Figures S1 and S2. [file aem.01810-25-s0001.docx]

**Supplementary information**

Table S1: Strains used in this study

| **Strain** | **Select Characteristics** | **Reference** |
| --- | --- | --- |
| *Escherichia coli* | | |
| DH5α | High-efficiency for cloning | New England Biolabs |
| K12 | Standard wild-type strain | (Lederberg and Tatum, 1946) |
| BL21-DE3 |  | ATCC |
| XL1-Blue |  | ATCC |

Table S2: Primers and DNA used in this study

| **Primer** | **Sequence** |
| --- | --- |
| TnGBP-5'NdeI.s | ggtcattgtCATatgCTCACCATCGGTGTCATCGGAAAATCC |
| TnGBP-3'NheI.a | TTCTGGCTGGCTAGCGAATTTTATCGGGATTCCGAGTTCCTCC |
| Univ-NdeI-5'.s | CTGCCCAGCCGGCGATGCATATG |
| Univ-NheI-3'.a | GGCGAGTTCTGGCTGGCTAGC |

**Table S3: Analysis of the PBP-fluorophore conjugates**

| **Protein** | **Mutation** | **Fluorophore** | **% Change** | **Ligand** | **Kd (µM)** |
| --- | --- | --- | --- | --- | --- |
| **TnGBP** | 131C | IANBD | +480 | Glucose | 11 |
|  | 131C | Alexa488 | +45 | Glucose | ND |
|  | 131C | Coumarin | +29 | Glucose | ND |
| **ABP** | 253C | IANBD | +48 | Arabinose | 770 |
|  | 253C | Alexa488 | +12 | Arabinose | 28 |
| **TteRBP** | 135C | Acrylodan | +20 | Ribose | 44 |
| **EBP** | 126C | Coumarin | -56 | Glutamate | 6410 |


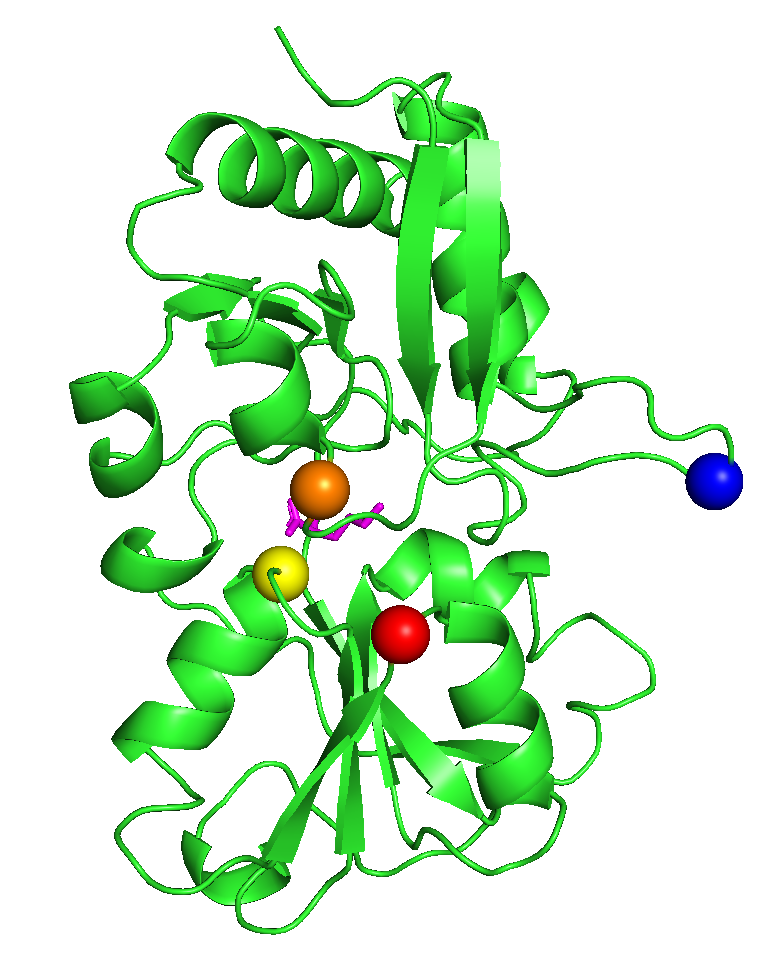


Figure S1: LAOBP in the ornithine-bound form (PDB code: 1LAH). Four cysteine mutants were constructed to attach thiol-reactive fluorophores at positions experiencing large changes in conformation between bound and unbound forms. The positions are represented by spheres: 22C (Blue), 54C (Orange), 120C (Yellow), and 141C (Red). Ornithine bound to the protein is shown in magenta.


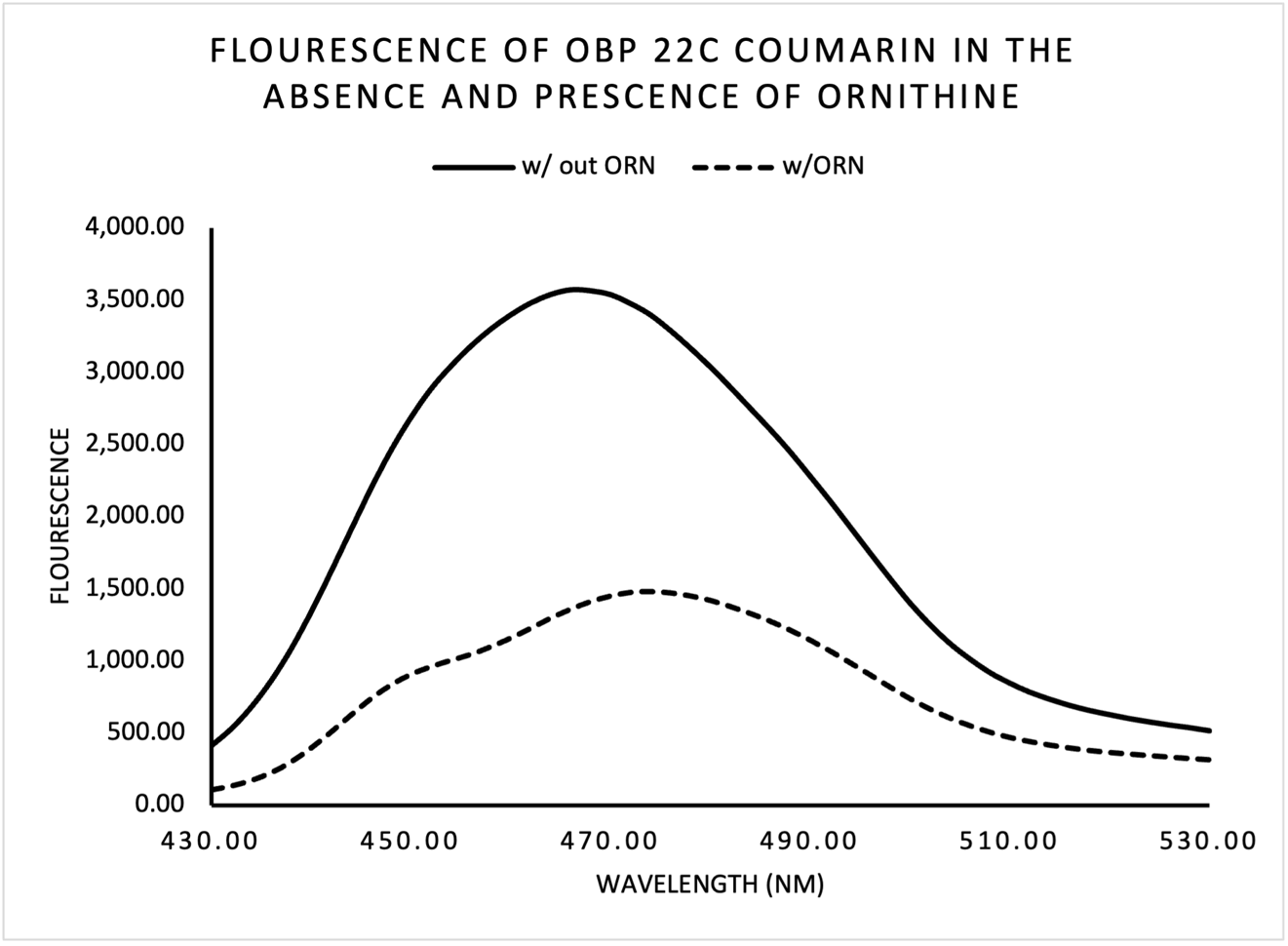


Figure S2: Change in fluorescence of the LAOBP-22C Coumarin conjugate upon addition of ligand. Solid line: no ligand, dashed line: 1mM Ornithine


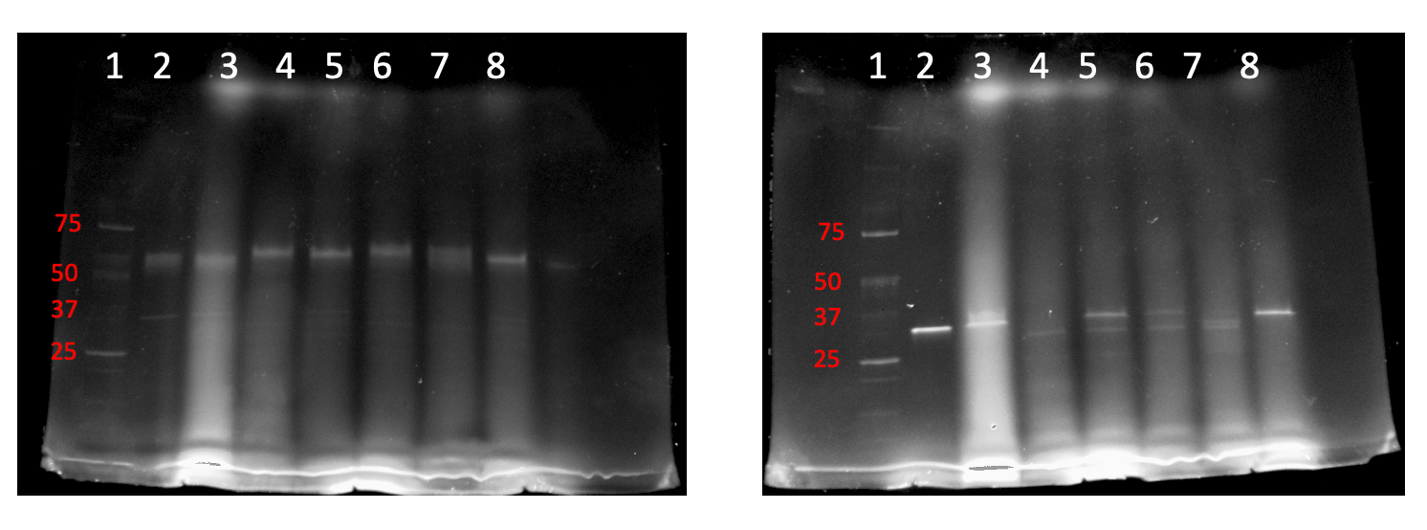


Figure S3: Gel electrophoresis analysis of the stability of the biosensors after incubation with different bacterial species in culture for 24 hours. Left gel: TnGBP, Right gel: LAOBP. A Bio-Rad gel imager was used to detect the fluorescently labeled biosensor in each sample. In each gel, the lanes are as follows: 1. MW standard, 2. Pure protein, 3. Pure protein in LB with no bacteria, 4 – 8: supernatants after 24 hours of cultures of *P. aeruginosa, S. aureus-*GBP, *E.coli*, *E. faecalis, and C. striatum.* For TnGBP (left gel), a single band corresponding to the size of a TnGBP dimer (66 kDa) persists in all cultures. For LAOBP (right gel), two bands are visible, one corresponding to the full-length protein (MW: 28.6 kDa), and smaller band of a truncated version corresponding to the MW weight of the protein after cleavage of the C-terminal HSV and His tag (MW:26.2 kDa).
